# A novel ToF-SIMS operation mode for sub 100 nm lateral resolution: Application and performance^☆^

**DOI:** 10.1016/j.apsusc.2013.10.177

**Published:** 2014-01-15

**Authors:** Markus Kubicek, Gerald Holzlechner, Alexander K. Opitz, Silvia Larisegger, Herbert Hutter, Jürgen Fleig

**Affiliations:** Institute of Chemical Technologies and Analytics, Vienna University of Technology, Getreidemarkt 9, A-1060 Vienna, Austria

**Keywords:** ToF-SIMS, Oxygen isotope analysis, Lateral resolution

## Abstract

A novel operation mode for time of flight-secondary ion mass spectrometry (ToF-SIMS) is described for a TOF.SIMS 5 instrument with a Bi-ion gun. It features sub 100 nm lateral resolution, adjustable primary ion currents and the possibility to measure with high lateral resolution as well as high mass resolution. The adjustment and performance of the novel operation mode are described and compared to established ToF-SIMS operation modes. Several examples of application featuring novel scientific results show the capabilities of the operation mode in terms of lateral resolution, accuracy of isotope analysis of oxygen, and combination of high lateral and mass resolution. The relationship between high lateral resolution and operation of SIMS in static mode is discussed.

## Highlights

• A novel ToF-SIMS mode and its performance and application is described.
• CBA mode allows measurements with sub 100 nm lateral resolution.
• Adjusting the primary ion current is possible for accurate oxygen isotope analysis.
• 4 application examples show novel or improved scientific results using CBA mode.

## 1 Introduction

Time of flight-secondary ion mass spectrometry (ToF-SIMS) has become a very popular technique for obtaining well resolved elemental and isotopic maps in two or three dimensions [1], [2], [3], [4]. While SIMS is a very sensitive technique for qualitative analysis, a quantitative elementary analysis from secondary ions is often not possible due to the complex sputter and ionization processes and matrix related differences in secondary ion intensities [5], [6], [7]. Isotopic analysis of a single element by SIMS is usually less troublesome. Even there, nonlinearities of the detection system or mass fractionation can account for errors in the determination of isotope fractions. Mass fractionation can occur for H and D [8] and is typically only a minor problem for elements with higher mass such as oxygen [9], [10]. Detection related errors can occur for all elements: a bad signal/noise ratio for minority isotopes and detector saturation/dead time effects as well as ion interaction in case of majority isotopes can cause serious problems for determining correct isotope fractions [11], [12], [13], [14]. The isotope analysis of oxygen with ToF-SIMS which is a central issue of this paper is of special interest for functional oxides and enables gathering thermodynamic and kinetic parameters of oxygen exchange and ion transport in these materials. Several techniques for analyzing tracer distributions are employed, such as depth profiling [15], [16], laterally resolved analysis of electrochemically active zones [17], [18] or of angle polished depth profiles [19], and also combined analysis for experiments requiring 3D data [20].

In Ref. [13] we introduced a novel ToF-SIMS operation mode, called “collimated burst alignment” (“CBA”) mode, which was optimized for oxygen isotope analysis. Its main features are an improved lateral resolution, accuracy of isotope fractions and adjustability of primary ion currents. Mass spectra were analyzed in detail in order to understand reasons of improved characteristics, particularly of improved accuracy of isotope fractions.

In this contribution we show several application examples with novel results obtained by using the CBA mode to demonstrate its excellent performance and capabilities for investigating scientific problems in surface science and materials research. Although isotope analysis of oxygen is primarily discussed, the novel operation mode is also adjustable for isotope analysis of other elements and applicable as imaging mode with high lateral resolution. It is further shown that the higher ion currents of the CBA mode compared to other imaging modes still retain the possibility of measuring with reasonable mass resolution in burst mode.

## 2 Methods

### 2.1 Instrumental details

Time of flight secondary ion mass spectrometry was performed on a TOF.SIMS 5 (ION-TOF, Germany) instrument. 25 kV Bi^+^ and Bi_3_^++^ were used as primary ions in different operation modes. The novel CBA mode and the related CBA-burst mode are compared to the BA and BA-burst mode. In both burst modes, 8 ion pulses were analyzed. Areas of 12 μm × 12 μm to 150 μm × 150 μm were investigated using a raster of 256 × 256 or 512 × 512 measured points. Negative secondary ions were analyzed and detailed information on the settings is provided with the particular application examples. For depth profiling and ablation of the surface, 2 kV Cs^+^ ions (500 μm × 500 μm, ca. 120 nA) were employed. For charge compensation, a low energy electron flood gun (20 V) was used.

### 2.2 Conventional ToF-SIMS operation modes

**Fig. 1:**
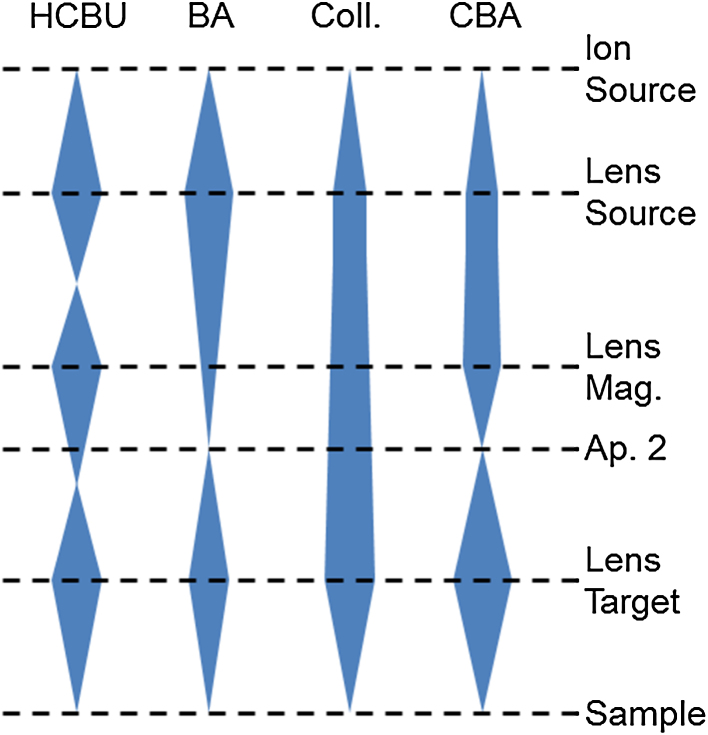
Schematics of beam guidance in several TOF.SIMS 5 operation modes.

The 25 kV primary ion column in TOF.SIMS 5 instruments has 3 lenses, enabling operation with different primary ion beam guidances. Three main operation modes are suggested by the manufacturers. High current bunched (HCBU) mode enables to measure with high currents and high mass resolution but very low lateral resolution. Burst alignment (BA) mode is a versatile mode, allowing reasonable lateral resolution (∼250 nm) at moderate primary ion currents. By operating the BA mode in the so called burst mode, the usually low mass resolution (*m*/Δ*m* ∼ 200) can be improved to *m*/Δ*m* > 6000 at the cost of lower currents. The collimated mode is an imaging mode, allowing a lateral resolution of about 100 nm at very low currents and low mass resolution. Application of the burst mode to increase mass resolution is impracticable due to motion of beam blanking (close to Aperture 2). As collimated mode does not have a crossover there, the lateral resolution would diminish. The important characteristics of these operation modes are summarized in Table 1 and respective beam guidances are outlined in Fig. 1.

**Table 1:** Primary ion gun operation modes on a TOF.SIMS 5 instrument using a 25 kV liquid metal ion gun (LMIG). Lens Source values are given as in operating software (FPanel), relative to extractor voltage.

Operation mode	HCBU	BA	Collimated	CBA
Lens Source (Extractor 9 kV)	∼3150 V	∼3300 V	∼3900 V	∼3750 V
Lens Mag	∼14.8 kV	0 V	0 V	12–13 kV
DC-current	∼15 nA	0.4–0.7 nA	50 pA	70–100 pA
Crossovers	2	1	0	1
Lateral resolution (lr)	2–10 μm	∼250 nm	∼100 nm	Bi^+^: 96 < lr < 136 nm Bi_3_^++^: 68 < lr < 96 nm
Mass resolution Burst	∼11,000 ×	Unit (∼200) >6000	Unit (∼200)	Unit (∼200) >6000

### 2.3 CBA mode

The notation “collimated burst alignment” (CBA) mode was chosen for the novel operation mode as it shares characteristics with both the collimated mode and the BA mode. In the upper part of the ion gun, the beam is almost parallel, very similar as in the collimated mode. In the lower part of the ion gun, the beam is focused into a crossover (at Aperture 2) before it is focused on the sample, exactly as in BA mode (cf. Fig. 1).

For adjusting the CBA mode, we suggest to start from the BA mode. First, the voltage on Lens Source (i.e. the lens closest to the Bi-emitter, cf. Fig. 1) is decreased (increased in ION.TOF FPanel software). Consequently, the beam focus is widened and thus the beam becomes more parallel. Second, the voltage on Lens Magnification is also increased until the beam is focused into a crossover at Aperture 2 (as in BA mode). In the lower part of the column, the beam is then focused on the sample with similar settings of Lens Target as in BA mode. Everyday adjustments for measurements are therefore almost identical to those of the BA mode. More details on the adjustment and operation of the CBA mode is given in Ref. [13]

As the voltages of Lens Source and Lens Magnification can be very finely controlled, the CBA mode is almost continuously adjustable from the BA mode. Increasing both lens voltages chiefly means trading a lower primary ion current for increased lateral resolution. This reduced current, however, is not necessarily a disadvantage for measurements; in case of oxygen isotope analysis for example, O^−^ has a very high secondary ion yield and the reduction of the primary ion current can even be necessary to avoid ion interaction and detector dead time effects and to maintain the accuracy of the measured oxygen isotope fractions. This requirement is much better met in CBA mode compared to the BA mode. The correlations of dead time effects, integration times, Poisson correction and accuracy of isotope fractions in CBA and BA mode are explicitly described elsewhere [13].

Regarding the adjustability of the CBA mode, the values of currents and lateral resolution given in Table 1 are values optimized for measurements on the functional oxides employed in this study. However, using lens voltages between the values given for BA and CBA mode could be advantageous for measurements in other systems, leading to ion currents and resolution values between those given in Table 1 for BA and CBA. Using the CBA mode as high resolution imaging mode also enables to measure with simultaneously high lateral and mass resolution in burst mode as demonstrated in Section 5.

One disadvantage of the CBA mode is the slightly higher aperture angle of the beam at the target compared to the BA mode (resulting from the beam being broader at Lens Target). Although the differences are not severe, this leads to a lower depth of focus, and consequently makes the exact adjustment of the sample height important in CBA mode. It should be mentioned, though, that depth of focus is of minor significance for ToF-SIMS measurements, as height differences and high sample roughness lead to unwanted shading or geometry related artifacts and have to be avoided anyway [21], [22].

## 3 Measurements making use of the high lateral resolution

### 3.1 General remarks on lateral resolution and static SIMS

Lateral resolution is an important characteristic not only for ToF-SIMS, but for all imaging techniques. The term resolution originates from optical microscopy [23] and today lateral resolution is defined in ISO 22493:2008 as “the minimum spacing at which two features of the image can be recognized as distinct and separate” [24]. However, several different approaches are common in the SIMS community to measure values of primary ion beam quality (such as sharpness) that are then called “lateral resolution” as pointed out by Senoner et al. [25]. In the same contribution, the coherences of the point spread function of the primary beam and image resolution, and the role of contrast and noise for measuring with a certain lateral resolution are described in detail. Further, a method to determine the lateral resolution is suggested [25]. As different measurement methods are used in the SIMS community and many of them do not meet the ISO definition, values for lateral resolution are often not directly comparable. For determination of the lateral resolution of the CBA mode, the method and criterion suggested by Senoner et al. [25] was employed. Lateral resolutions (lr) of 96 nm < lr < 136 nm for Bi_1_^+^ and 68 nm < lr < 96 nm for Bi_3_^++^ were determined on a BAM-L200 certified reference sample with details on the measurements and evaluation of lateral resolution in CBA mode being given in Ref. [13].

Static SIMS and dynamic SIMS are two different methods and approaches for investigations with SIMS. The main difference is the current of primary ions and the damage inflicted by them to a target surface. Dynamic SIMS works with high currents and accepts damage of the target surface and also uses this ablation for depth profiling. Static SIMS uses much lower currents in order to keep the damage of a target area small. Measuring for a certain time on the same surface area should therefore yield the same undisturbed signal, if the ion dose per area is below the so called “static limit” [26]. This limit is about 10^13^ ions per cm^2^ for inorganic materials [3], [27] and ∼10^12^ cm^−2^ for organic materials [4], [27] and depends on the sputter yield of the primary ion, ion energy, target surface, etc. (a typical surface consists of ∼10^15^ atoms cm^−2^). By improving the lateral resolution and thereby reducing the target area of the primary ions it becomes more difficult to measure in static mode. The connection becomes clear when imagining a primary ion source with ideal (atomic) lateral resolution. For realizing this lateral resolution, all surface atoms have to be hit by primary ions and removed to be detected as secondary ions. However, this type of operation is clearly dynamic SIMS.

A quick calculation for a typical measurement in the CBA mode, assuming a Bi^+^ primary ion current of 0.04 pA, a 10 μm × 10 μm area, and 10^13^ ions cm^−2^ as static limit, allows a measurement time of about 40 s until reaching the static limit. By this calculation we see that CBA mode can still be operated within the static limit condition. However, measuring with lateral resolutions below 100 nm bears the risk of violating the static limit without being aware of it. A further increased lateral resolution to values below 20 nm, as shown for state-of-the-art ToF-SIMS instrumentation [28] might even be incompatible with the limits of static SIMS.

Also in part of the following measurements with high lateral resolution the static limit was violated in favor of higher signal intensity. This, however, is not problematic for validity and resolution of the oxygen isotope distribution images since no dynamic processes or organic materials were investigated in this contribution.

### 3.2 ^18^O incorporation into YSZ via Pt electrodes

**Fig. 2:**
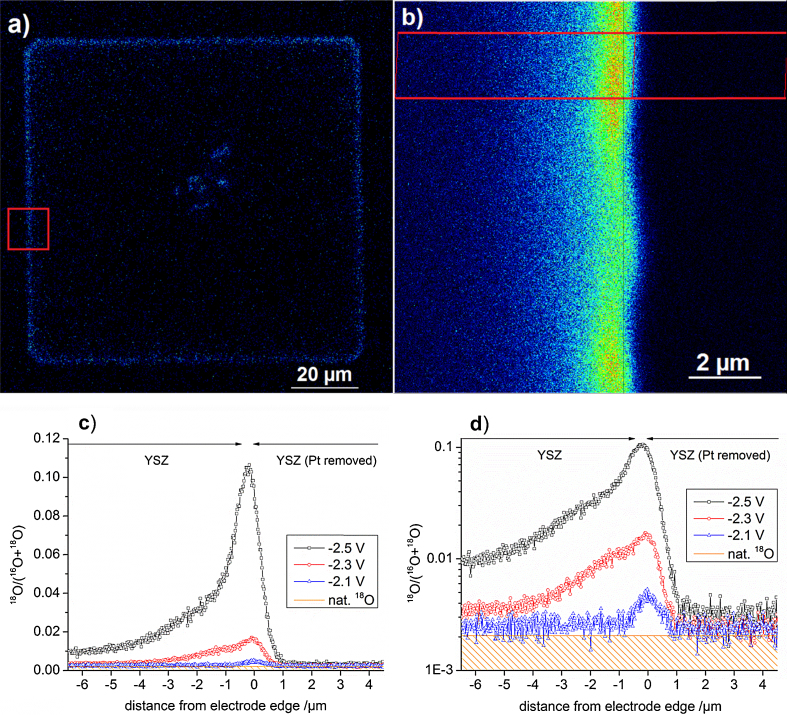
(a) Overview image of (1 1 1) YSZ after ^18^O incorporation with −2.5 V set voltage (Pt electrode removed); (b) detail image of a former Pt|YSZ phase boundary (highlighted area in a); (c and d) linear and logarithmic plot of the ^18^O fraction in the line scan/integration area in (b) and of equivalent experiments using lower overpotentials. The overview image (a) is undersampled and does not display the full lateral resolution possible with CBA-mode. (For interpretation of the references to color in this figure legend, the reader is referred to the web version of the article.)

The oxygen reduction reaction on platinum and incorporation of oxygen into solid electrolytes is a model reaction for many applications (fuel cells, sensors, catalysis). Here we present SIMS measurements for visualizing the electrochemically active sites of the model system Pt(O_2_)|yttria stabilized zirconia (YSZ) by means of ^18^O tracer incorporation. The tracer experiments were conducted at ∼290 °C and ^18^O was incorporated into YSZ (1 1 1) single crystals (9.5 mol% Y_2_O_3_) on square shaped (100 μm × 100 μm) Pt thin film electrodes by applying a cathodic dc voltage. The tracer (^18^O_2_ gas) was locally supplied to the Pt microelectrode by a quartz capillary and different voltages were used (between −2.1 and −2.5 V); for experimental details the reader is referred to Ref. [17]. After ^18^O incorporation and before SIMS measurements, the Pt electrodes were removed from YSZ by etching in hot nitro-hydrochloric acid. This important step guarantees a surface without height differences for SIMS analysis and additionally avoids measurement artifacts caused by distortions of the extraction field of secondary ions by areas with different electrostatic charge (Pt, YSZ) during the dynamic sputter process. Similar measurements have already been reported in literature [17], [29], [30], [31], [32], [33], but here we show that applying the novel CBA measurement mode (and hereby improving the lateral resolution) helps unveiling further details of the oxygen incorporation process as shown in Fig. 2.

Measurements were performed in CBA mode with Bi^+^ primary ions. ^18^O distribution images of 130 μm × 130 μm and 12 μm × 12 μm size were measured with a resolution of 512 × 512 and 256 × 256 pixels, respectively, and detail images were integrated over 900 image scans. The overview image is undersampled and does not display the full lateral resolution possible with CBA-mode. Line integration within the detail images was used to investigate the lateral distribution of the active area for oxygen incorporation. The integration area was chosen on a straight Pt edge to minimize the blurring effect of the electrodes waviness – cf. the rectangular box in Fig. 2b. The lateral profiles resulting from line integration are depicted in Fig. 2c and d in linear and logarithmic scale, respectively. These images show the tracer fraction as a function of lateral position.

Three different reaction sites for oxygen incorporation and exchange can be distinguished in the lateral profiles in Fig. 2c and d:

(i) Beneath the Pt electrode, caused by oxygen incorporation at the Pt|YSZ interface [17], [34]. Grain boundaries in Pt are suggested to offer a pathway for oxygen through the gas tight thin film. Since the size of Pt grains is only about 100 nm and the differences in ^18^O fractions are very low, the grain boundaries cannot be resolved in these measurements. The amount of tracer incorporated within the given time (i.e. the faradaic current during the incorporation experiment) is in acceptable agreement with data from electrochemical measurements [34], [35].
(ii) The increased tracer incorporation in a zone close to the triple phase boundary (TPB) extending along the free YSZ surface was already discussed to proceed via an electrolyte surface path [17], [35]. This means O_2_ adsorbs on the electrolyte and electrons for reduction are supplied by YSZ. At the given temperatures (∼290 °C) the Pt electrodes are electrochemically highly blocking and stoichiometry polarization takes place in YSZ upon high cathodic polarization. This leads to an increased electron concentration within the electrolyte in the vicinity of the electrode, and caused the strong increase in oxygen incorporation rate on the YSZ surface close to the TPB.
(iii) On YSZ surface regions far enough from a polarized electrode to be not affected by stoichiometry polarization, tracer incorporation proceeds via a “classic” entropy driven oxygen tracer exchange process. This process could only be monitored in case of the lowest polarization (*η* = −2.06 V) on the YSZ part far away from the electrode edge (blue curve in Fig. 2c and d, *x*-scale −6 to −2 μm).

**Fig. 3:**
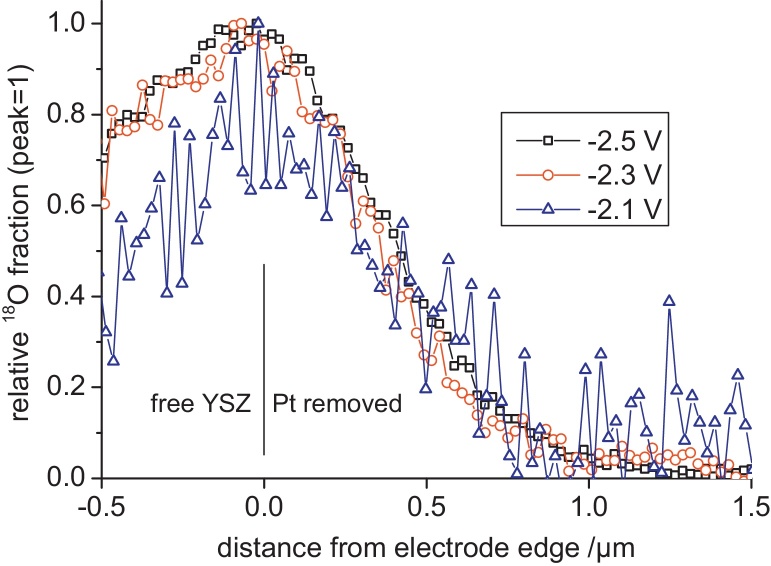
Extension of oxygen incorporation beneath a Pt electrode. Tracer fractions related to the respective maximum tracer fractions are plotted for different set voltages.

The oxygen incorporation on stoichiometry polarized YSZ close to the TPB (path ii) causes a strongly asymmetric lateral profile. In Ref. [17] this asymmetry could only be resolved for very high cathodic polarization. The improved resolution of the CBA mode now allows us to visualize this asymmetry also for lower cathodic overpotentials. The broadening along the free YSZ surface strongly depends on the polarization, in agreement with the strongly voltage dependent electron concentration due to stoichiometry polarization in YSZ. A plot of the relative tracer fractions close to the Pt|YSZ edge is shown in Fig. 3. The sharpness of the ^18^O drop beneath (removed) Pt is between 400 and 500 nm for all electrode polarizations (84–16% intensity drop).

In the present study the sharpness is limited by the following effects: diffusion of tracer in YSZ during the experiment (about 280 nm diffusion length for given time/temperature) [36], lateral resolution of the beam (∼100 nm), waviness of the electrode edge in the integration area (100–200 nm, estimated from SEM images) and slight beam shifts during ToF-SIMS measurements. The experimentally found decay length of the lateral tracer profile beneath Pt of 400–500 nm can thus be concluded to be predominantly caused by other factors than by a true broadening of the electrochemically active zone along the Pt|YSZ interface. Only the broadening along the free YSZ surface reflects an electrochemical effect. Consequently we can conclude from these new, highly resolved measurements, that the active zone of oxygen incorporation on Pt|YSZ upon high cathodic polarization shows a very asymmetric extension along the free YSZ surface and virtually no extension beneath the Pt electrode.

### 3.3 Oxygen diffusion in oxidized steel

**Fig. 4:**
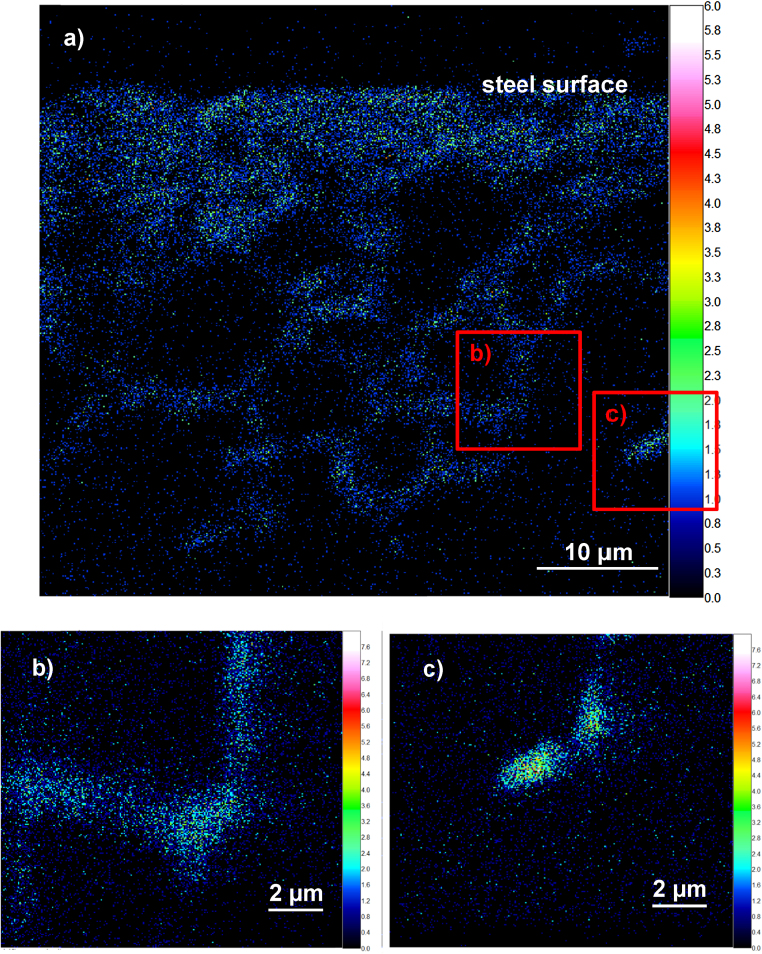
(a) ^18^O^−^ lateral map visualizing tracer diffusion in an angle polished, oxidized steel sample (undersampled); (b and c) ^18^O^−^ detail images of the areas depicted in (a) displaying the achievable lateral resolution with CBA-mode.

As a second example demonstrating the important role of lateral resolution for unveiling structural details, we present measurements on steel. Model steel samples were first oxidized with oxygen of natural isotope distribution. In a second step, an ^18^O tracer exchange experiment was performed in order to study the diffusion of oxygen in the oxidized grain boundaries. As tracer diffusion lengths of several μm were realized, samples were angle-polished in order to transform tracer depth profiles into lateral profiles. ToF-SIMS measurements were performed in CBA mode with Bi_3_^++^ primary ions. An overview image of the ^18^O secondary ion distribution of 50 μm × 50 μm measured with 512 × 512 pixels and integrated over 200 images is shown in Fig. 4a. Detail images of 12 μm × 12 μm were measured with 256 × 256 pixels and integrated over 300 images (Fig. 4b and c).

The visualization of diffusion paths along grain boundaries and the determination of the decrease of tracer concentration with depth are possible from the overview image. By applying the CBA mode in small areas and increasing the integration time, it was possible to resolve more details of grain boundary regions. An inhomogeneous ^18^O distribution is discernible along grain boundaries (Fig. 4b) in some areas, even point-type ^18^O enrichments become visible (Fig. 4c). It is found that triple junctions show a higher ^18^O concentration than boundaries between 2 grains (Fig. 4b). These observations suggest that quasi 1D oxygen diffusion along triple junctions may play an important role also in integrated diffusion depth profiles. By investigating a cutting-plane, these filaments typically lead to point-type ^18^O enrichments as discernible in Fig. 4c.

**Fig. 5:**
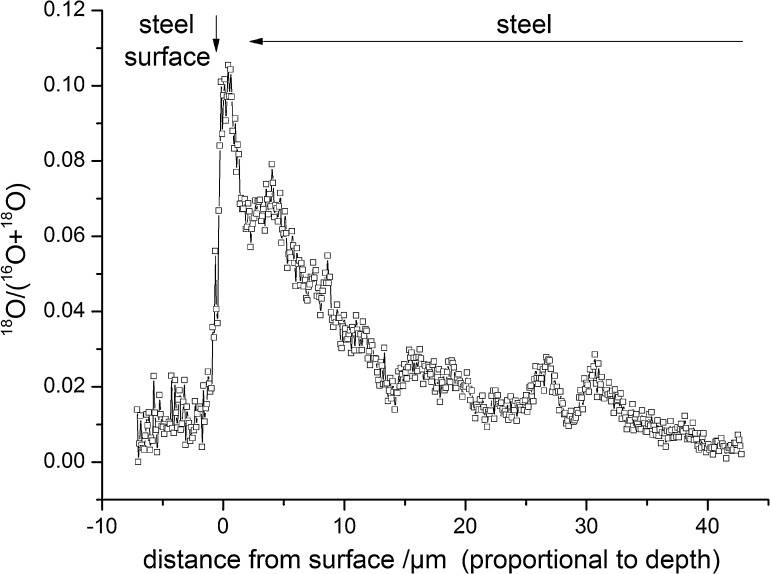
^18^O tracer depth profile extracted via line integration from the measurement also shown in Fig. 4a.

Modeling of such a system to extract diffusion coefficients is therefore very complex. The more important are highly resolved images to discern or even quantify these effects in detail. Only considering integrated depth profiles (as shown in Fig. 5) cannot provide these detailed information and misinterpretation of such depth profiles can be avoided.

## 4 Mass resolution in CBA-burst mode

**Fig. 6:**
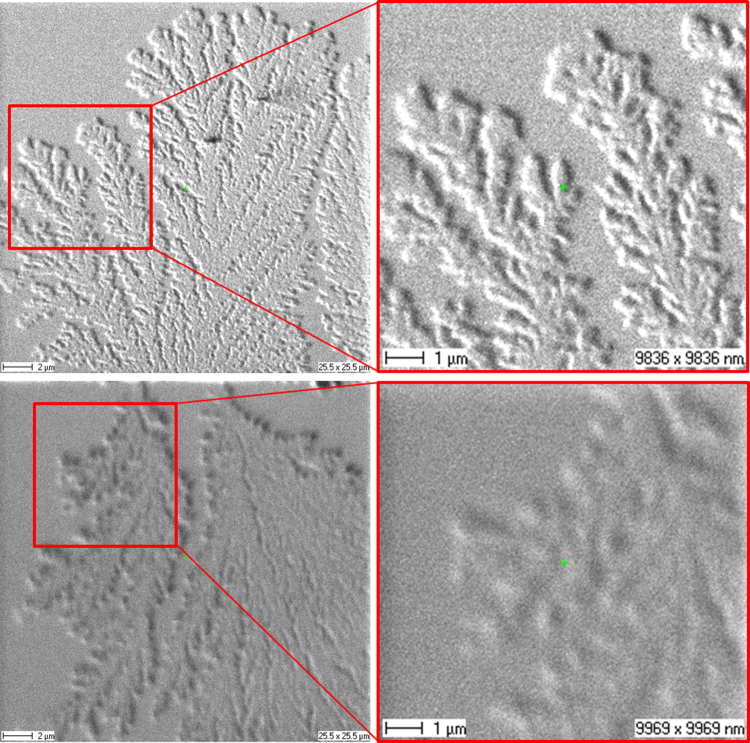
Measurements of copper dendrites on silicon nitride. Differences in image resolution achieved in CBA mode (top) and BA mode (bottom) are shown for secondary electron overview images (left) and detail images (right).

The corrosion of copper in humid environment is of great interest for electronics and semiconductor technology. Migration and diffusion processes of copper between differently charged electrodes are examined in this example under wet conditions and electric bias. Beside the well-known growth of copper dendrites from the cathode to the anode, different corrosion products of copper (e.g. copper salts, copper oxides) are partially spread over the surface [37] during the corrosion process. Secondary electron images of copper dendrites on silicon nitride (Fig. 6) demonstrate the higher lateral resolution obtainable with the CBA mode compared to the BA mode. Among the corrosion products are also sulfur-containing compounds. The majority isotope of sulfur ^32^S, however, has a mass interference with ^16^O_2_ which is often present in significant intensities. Hence, both a high mass resolution and a high lateral resolution are required to get insight into these corrosion phenomena.

**Fig. 7:**
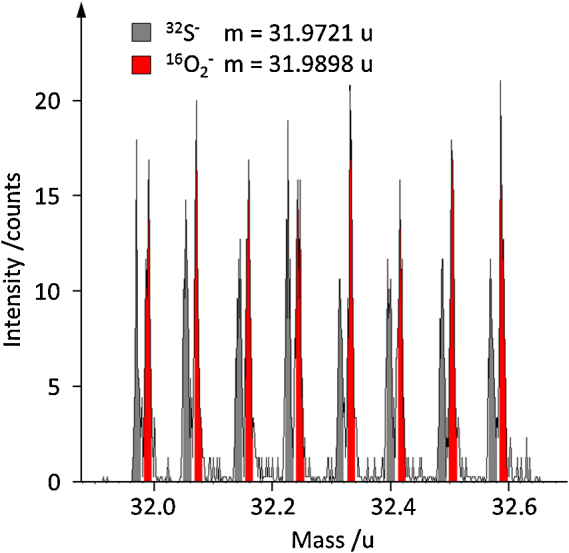
Mass spectra obtained in CBA burst mode with 8 bursts allowing the separation of ^16^O_2_^−^ and ^32^S^−^. The mass scale is only valid for the first burst.

Typical ToF-SIMS imaging modes only have unit mass resolution and are thus unable to separate ^16^O_2_ and ^32^S. In BA and CBA mode it is possible to reach a higher mass resolution by operating them in burst mode. By chopping the primary ion beam to packages of ∼1.5 ns in burst mode it is possible to reach mass resolutions *m*/Δ*m* higher than 6000. The major downside of the burst mode is the strong loss of intensity (ca. factor 30). Intensity can be improved again by applying several consecutive bursts (shown for 8 bursts in Fig. 7) given that there are no mass interferences in the mass range claimed by the additional bursts. Fig. 7 also shows that the separation of ^32^S^−^ and ^16^O_2_^−^, which requires a mass resolution of *m*/Δ*m* > 1800, can be easily done in CBA burst mode.

**Fig. 8:**
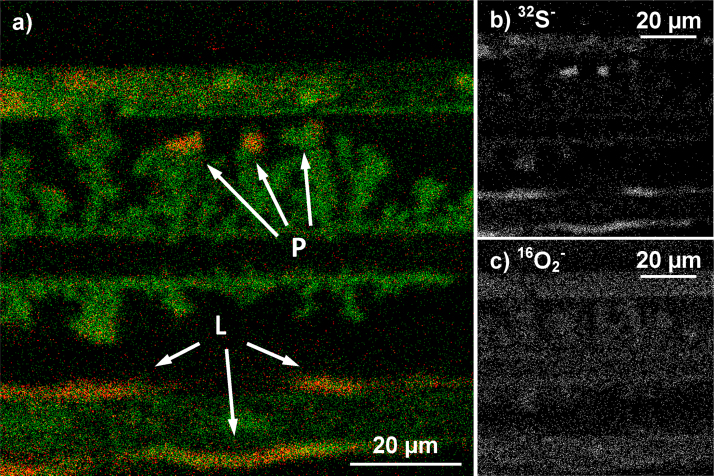
(a) Overlay image of ^16^O^−^ (green) and ^32^S^−^ (red) showing point-shaped (P) and line-shaped (L) accumulation of sulfur; (b) ^32^S^−^ image of the same area separated in CBA-burst mode from the ^16^O_2_^−^ distribution image shown in (c). (For interpretation of the references to color in this figure legend, the reader is referred to the web version of the article.)

This mass separation makes imaging of the sulfur distribution possible. In Fig. 8a an overlay image of ^16^O^−^ (green) and ^32^O^−^ (red) is shown. While the oxygen image resembles the dendrite structures of copper, high intensities of sulfur are only found in several places. The images in Fig. 8 were created from a measurement in CBA mode of 85 μm × 85 μm measured with 1024 × 1024 pixels and integrated over 25 images. Point-like sulfur enrichment is found at the tips of copper dendrites, while line-shaped enrichment is visible at both edges of the anodes. The distribution images of both mass 32 signals, ^32^S^−^ and ^16^O_2_^−^ are displayed in Fig. 8b and c showing that the enrichment in the S-signal has no counterpart in the ^16^O_2_^−^ distribution image and further verifying that mass separation was successful.

## 5 Accuracy of isotope fractions and its role in isotope exchange depth profiling

The accuracy of isotope fraction measurements is especially important for geologic samples, but it also plays a role for tracer diffusion experiments on functional oxides. Oxygen has three stable and naturally occurring isotopes, ^16^O as majority isotope (∼99.76%) and ^17^O (∼0.04%) and ^18^O (∼0.2%) as minority isotopes. The natural isotope distribution of oxygen varies slightly in water, atmosphere and lithosphere. The values for the natural isotope fraction ^18^O/^16^O most commonly used in literature are from National Institute of Standards and Technology (NIST) 2.05(14) × 10^−3^ and the Vienna Standard Mean Ocean Water (VSMOW) definition of International Atomic Energy Agency, ^18^O/^16^O = 2.00520 × 10^−3^. Typically in air, water and oxygen containing solids, oxygen isotope ratios within this range of variation are found. However, it should be noted that bottled oxygen, which is often used in preparation or annealing steps, usually does not have natural isotope distribution. Depending on preparation and purity, often significantly higher ^18^O/^16^O ratios up to 5 × 10^−3^ can be expected in bottled oxygen [38].

### 5.1 Isotope exchange depth profiling on functional oxides

Isotope exchange depth profiling (IEDP) is a methodically simple approach. First, tracer depth profiles are established by tracer gas exchange at elevated temperatures, then the diffusion profiles are frozen-in at room temperature and measured, e.g. with ToF-SIMS. When using oxygen tracer enriched gas, kinetic parameters such as the oxygen exchange coefficient k* and the tracer diffusion coefficient D* can be obtained. In a typical IEDP experiment oxygen exchange is investigated on a 200 nm thin film of La_0.6_Sr_0.4_CoO_3−*δ*_ (LSC), a mixed ionic and electronic conductor, on 500 μm yttria stabilized zirconia (YSZ, 9.5 mol% Y_2_O_3_). Thermal oxygen exchange was performed for 5 min in 200 mbar ^18^O_2_ (97.1% enriched) at 376 °C. The depth of the film was determined from sputter coefficients based on reference measurements analyzed by digital holography microscopy.

**Fig. 9:**
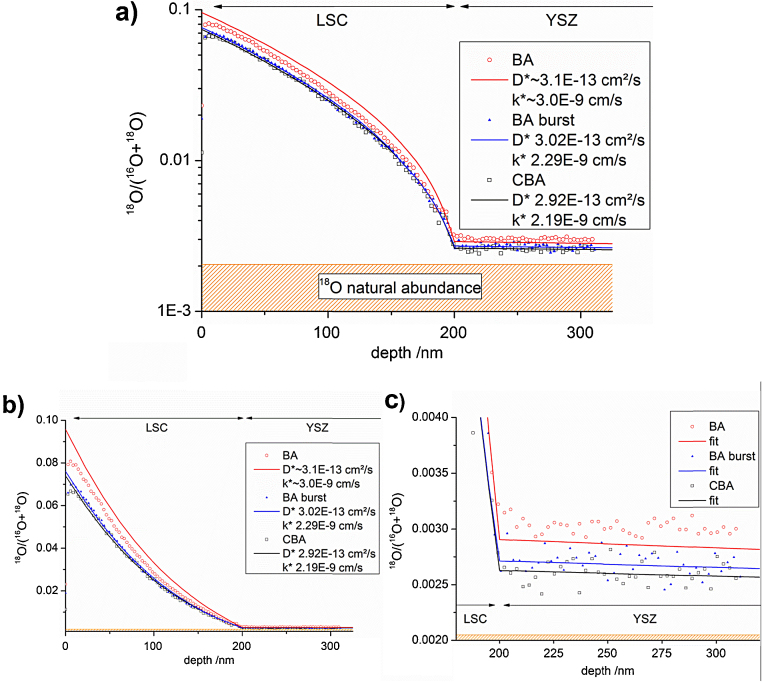
^18^O isotopic fraction in (a) logarithmic and (b) linear plot. (c) Magnification of (b) showing the different ^18^O fractions in YSZ.

**Fig. 10:**
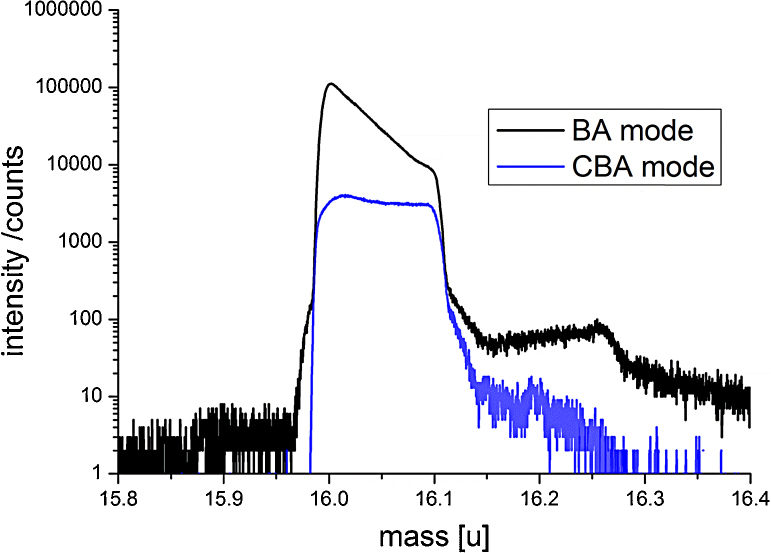
Mass spectra of the ^16^O^−^ signal measured with ∼50 ns pulse width in BA and CBA mode. In BA mode, detector saturation effects as well as intensity preceding and succeeding the main signal as result of ion interactions are visible.

SIMS measurements were then performed in three different modes with Bi^+^: in CBA mode, in BA-burst mode with integration as described by De Souza et al. [39], and in BA mode. Different isotope fraction profiles are determined by these 3 methods as shown in Fig. 9 and reproduced in two measurements each, at the following primary ion currents: BA 0.375 pA, BA-burst 0.093 pA, CBA 0.045 pA. Parameters affecting the correct determination of oxygen tracer fractions are discussed in literature [11], [40], [41]. Essentially, the reason for the differences here can be found in the high ^16^O^−^ secondary ion intensity in BA mode. Although Poisson correction is applied for all measurements, in BA mode ^16^O^−^ counting by the detector is falsely too low (see also Ref. [13]). This is a result of saturation effects that are not sufficiently correctable by Poisson statistics and of interaction of secondary ions. Mass spectra ^16^O^−^ measured in BA and CBA mode are shown in Fig. 10. Because of the wrong counting of ^16^O^−^ and the correct counting of ^18^O^−^, a too high ^18^O tracer fraction is measured in BA mode. For the measurements in CBA mode, both isotopes intensities are within the linear counting regime of the detector, thus correct values of the isotope fractions can be expected.

In order to extract the parameters k* and D*, the profile was then modeled as 1D-diffusion problem in COMSOL Multiphysics 4.0 software with 2 fit parameters: (i) the surface exchange coefficient k* of LSC and (ii) the diffusion coefficient D* of LSC. The slope of the profile in YSZ is too small to allow determination of a reasonably accurate diffusion coefficient of YSZ. Therefore the diffusion coefficient of YSZ was taken from electrical reference measurements on YSZ single crystals at 376 °C (1.3 × 10^−10^ cm^2^/s) and not further varied. This value also corresponds well to data reported in literature [42]. Fitting functions agree very well with all measured data in CBA and BA-burst mode. The sharp edge at the LSC|YSZ interface simply reflects the step in diffusion coefficient, which is much larger in YSZ. Values of k* and D* are shown in Fig. 9 for the respective depth profiles.

For this example, we can extract values of k* = 2.19 × 10^−9^ cm/s and D* = 2.92 × 10^−13^ cm^2^/s from the CBA measurement. Measurement in BA-burst mode yield only slightly higher values of k* = 2.29 × 10^−9^ cm/s and D* = 3.02 × 10^−13^ cm^2^/s. For the same depth profile measured in BA-mode, the quality of the fit is considerably lower. This indicates that the changes of the deduced isotope fractions lead to a non-physical diffusion profile. In contrast to the profiles obtained with the other measurement modes, the front-part (LSC) of the diffusion profile or the back-part (YSZ) cannot be reproduced with one and the same data set of D* and k*.

Using a fit curve which overestimates the tracer fraction in LSC and underestimates the fraction in YSZ yields k* = 3.0 × 10^−9^ cm/s and D* = 3.1 × 10^−13^ cm^2^/s, and thus a significantly higher k* value which is a result of an erroneous isotope fraction measurement.

It can be concluded, that the correct determination of isotope fractions can only be performed in a certain intensity window of secondary ions, which depends on many parameters (isotopes analyzed, detection system, secondary ion yield, etc.). The CBA mode offers the possibility to adjust the primary ion beam current such that the secondary ion intensity is inside this window thus avoiding systematic errors resulting from either too high or too low secondary ion intensity.

## 6 Conclusions

The novel CBA operation mode for ToF-SIMS measurements was successfully applied to several scientifically relevant examples in materials research. By adapting the beam guidance from burst alignment mode, it is possible to achieve lateral resolutions below 100 nm at higher currents than in other imaging modes. Besides application as imaging mode, the CBA mode offers the possibility to adjust the primary ion beam currents over a wide range, making it ideal for isotope analysis. The higher lateral resolution of the CBA mode helped unveiling important details of electrochemically active zones of Pt|YSZ and fast diffusion paths in oxidized steel. Stepless adjustment of the primary ion beam current to lower intensities as in BA-mode can avoid detection non-linearity effects and allows measurement of correct isotope fractions. Measuring in CBA mode offers also the possibility to achieve both high lateral and a high mass resolution. This enabled detection of sulfur species on Cu dendrites despite the presence of ^16^O_2_ signal. Accurate isotope fraction determination in CBA mode was demonstrated to be important when analyzing diffusion coefficients and oxygen exchange coefficients, for example of LSC thin films on YSZ. It was also shown that it is possible to remain within the boundaries of static SIMS when measuring with CBA mode.
